# Reliability and validity of the Swedish version of the inventory of school attendance problems (ISAP)

**DOI:** 10.1007/s00787-024-02618-0

**Published:** 2024-11-28

**Authors:** Johan Strömbeck, David Heyne, Laura Ferrer-Wreder, Katarina Alanko

**Affiliations:** 1 Faculty of Arts, Psychology and Theology, Åbo Akademi University, Fabriksgatan 2, Åbo, 20500 Finland; 2 Magelungen Utveckling AB, Stockholm, Sweden; 3 School of Psychology, Deakin University, Burwood, Australia; 4 Department of Psychology, Stockholm University, Stockholm, Sweden

**Keywords:** School attendance problems, Assessment, Inventory of School attendance problems, Confirmatory factor analysis, Internal consistency, Convergent validity

## Abstract

School attendance problems (SAPs) are heterogenous in nature and thus warrant rigorous assessment prior to intervention. The Inventory of School Attendance Problems (ISAP) is a new instrument that supports a comprehensive assessment of SAPs. This study is the first evaluation of the Swedish version of the ISAP. A web survey comprising the ISAP, School Refusal Assessment Scale-Revised (SRAS-R), and the Strengths and Difficulties Questionnaire (SDQ) were administered to a Swedish community sample (*n* = 399) of secondary school students aged 12–16 years. We employed a cross-sectional design to test the factor structure of the Swedish ISAP using confirmatory factor analysis (CFA). Additionally, we evaluated internal consistency using Cronbach’s alpha and omega total, and we evaluated convergent validity by comparing the ISAP with subscales from the SRAS-R and SDQ. Factor analysis yielded support for a predicted 13-factor model. Despite the Chi-square test indicating a significant misfit, the remaining fit indices suggested an adequate model fit. In this sample, there was adequate internal consistency for most scales. Correlations between ISAP factors and related constructs were generally large and positive. Overall, the findings support the reliability and validity of the Swedish ISAP, indicating its potential as a valuable tool for understanding SAPs among students in community samples. Further research is needed to explore its applicability in clinical samples.

**Supplementary Information:**

The online version contains supplementary material available at 10.1007/s00787-024-02618-0.

School absence has been associated with anxiety disorders [1], depression [2], and an elevated risk of self-harm and suicide ideation [3]. In the early school years, absence has been linked with suboptimal literacy skills, lower grade point average, and increased absence during adolescence [1]. Furthermore, it can lead to long-term negative consequences, such as reduced educational attainment and greater economic difficulties [2]. Various risk factors contribute to school absence, such as anti-social behaviour and cognitions, a negative attitude towards school, limited parent involvement in school, poor student-teacher relationships [4], and an unfavorable school climate [5, 6]. In summary, school attendance problems (SAPs) are multifaceted, with diverse manifestations and determinants [7]. Therefore, a comprehensive assessment of a young person’s difficulties in attending school is essential to inform the development and implementation of effective interventions [8, 9].

Various instruments have been developed to better understand SAPs [10]. For example, the School Refusal Assessment Scale-Revised (SRAS-R) [11, 12] was designed to measure four functions of SAPs that are linked to positive and negative reinforcement. Another instrument, the Self-Efficacy Questionnaire for School Situations (SEQ-SS) [13], measures youths’ cognitions associated with school attendance. Additionally, the SChool REfusal EvaluatioN scale (SCREEN) [14] measures indicators of school refusal among adolescents. Each of these instruments measures a limited range of variables associated with SAPs, predominantly focused on the young person’s thoughts, feelings, and behavior. The field of school attendance lacks a measure that encompasses a broad range of factors potentially associated with SAPs. Furthermore, assessing the reasons behind absence should extend beyond the individual to encompass broader factors, such as the family and school environment.

The Inventory of School Attendance Problems (ISAP) was developed in Germany by Knollmann and colleagues [15], serving as an alternative to the SRAS-R for understanding SAPs. It addresses a broader range of factors associated with SAPs, including symptoms like anxiety and depression, relational problems, lack of motivation, and the young person’s experience of their current school. The ISAP measures both the quality and function of 13 factors related to SAPs. Here, ‘quality’ refers to the presence of symptoms, while ‘function’ pertains to the impact these symptoms have on school attendance. Each ISAP item consists of a single stem, such as ‘I’m afraid of exams’, and respondents are asked to rate the extent to which the item applies to them (i.e., as a symptom) and how much it influences their school attendance (i.e., as a function of absence). Importantly, the instrument was designed to measure the 13 factors in both youths who are non-attending and those who attend school but struggle with attendance. Knollmann and colleagues [15] suggest that this adaptability makes the ISAP well-suited for screening purposes, as it can identify factors associated with both emerging and established SAPs.

To gain insight into the construct validity of the ISAP, Knollmann and colleagues [15] relied on principal component analysis (PCA) conducted on data from a clinical sample of youths, which resulted in a 13-factor solution. The internal consistencies of the scales were acceptable (Cronbach’s alphas between 0.75 and 0.88), but some items demonstrated cross-loading. Specifically, 12 of the 48 items had cross-loadings of 0.30 or higher, with three items at or above 0.40. These findings indicate the need for further investigation using different samples and analytical methods.

While PCA is a useful tool in the early stages of scale development, it should be supplemented with more rigorous analytical methods. Confirmatory Factor Analysis (CFA) provides a way to test specific theoretical models of the scale’s underlying structure and is also valuable for evaluating the utility of a translated questionnaire in new contexts [16, 17]. In their PCA, Knollmann and colleagues [15] combined the symptom and function scores, even though the ISAP includes separate scales for these constructs. A more informative approach would be to test the factor structure of the symptom and function scales separately. This would enable researchers to explore potential differences in the associations between SAPs and symptoms on the one hand, as well as between SAPs and the functions of symptoms on the other hand.

To assess the ISAP’s convergent validity (referred to as construct validity in their study), Knollmann and colleagues [15] correlated the factor scores with subscales from German versions of the SRAS [18] and the Youth Self Report (YSR) [19]. They found large correlations between the ISAP’s internalizing scales and the YSR’s internalizing scales, as well as between the ISAP’s and YSR’s externalizing scales. Additionally, some ISAP scales showed large correlations with scales from the German SRAS.

Since its development by Knollmann and colleagues [15], the ISAP has not undergone further evaluation. Recognizing the instrument’s potential value for both researchers and practitioners, we developed a Swedish version and examined its reliability and validity in a more generalizable, community school-based sample. Specifically, we tested the 13-factor structure identified by Knollmann and colleagues for both the symptom and function scales, assessed the internal consistency of the scales, and evaluated the convergent validity of the Swedish ISAP. Our research questions were:

1. Can the 13-factor structure identified in the original clinical sample be replicated in a community school-based sample?
2. Do the Swedish ISAP scales demonstrate acceptable internal consistency?
3. Does the Swedish ISAP exhibit convergent validity with conceptually related scales, namely positive and negative psychological states and behaviors (measured by the Strengths and Difficulties Questionnaire [SDQ]), and the functions of school absence (measured by the SRAS-R)?

## Method

### Sample

From the initial sample of 414 students, 15 were excluded from the analyses for various reasons, including lack of written consent, missing age information, or being outside the study’s age range of 12 to 16 years. The final sample (Table 1) consisted of 399 students aged 12 to 16 years (*M* = 14.7, *SD* = 0.9; see Table 7 in the Supplementary Materials for age distribution), with 53% identifying as girls, 45% as boys, and 2% as another gender. Among the participants, 28% reported having special education needs, 15% reported depression-related problems, 29% anxiety, 17% ADHD or ADD, 8% autism spectrum disorder, and 1% reported having behavioral problems.

**Table 1:** *Participants characteristics*

	Typical school setting	Resource school	Student health team	Total
*N*	318	63	18	399
Gender % (Female/Male/Other)	55/43/2	40/60/0	56/39/6	53/45/2
Age – *M* (*SD*)	14.7 (0.9)	14.4 (1.0)	14.2 (1.0)	14.7 (0.9)
Age – Min-max	13–16	12–16	12–16	12–16
School absence ^a^*M* (*SD*)	2.3 (3.9)	4.0 (4.5)	9.0 (7.7)	2.9 (4.5)
SAP (%)	100 (31)	24 (38)	17 (94)	141 (35)

Note. *N* = number of subjects, *M* = mean, *SD* = standard deviation, Min = Minimum value, max = maximum value, SAP = School attendance problems

^a^ Self-reported number of whole days absent during the last 20 school days

### Procedure

We used a cross-sectional research design. Participants were recruited through the schools that were recruited via a webinar and e-mail outreach. Schools could opt to involve: (a) all students within the school, (b) students from one or more classes, or (c) individual students referred to the student health team^1^ due to SAPs. Thirteen schools participated, including three that involved the whole school (one of which was a resource school, which is a smaller school setting with more staff, serving children with special education needs), one school that involved a single class, and nine schools that recruited individual students. In total, 79.7% of students came from a typical school setting, 15.8% from a resource school, and 4.5% via the student health team.

Participating students completed a web survey. They had two options for responding: (1) in the school setting, or (2) through the student health team. In the school setting, students answered the web survey anonymously in the classroom during school hours, using either a school computer or their personal cell phone. In contrast, students responding through the student health team completed the survey individually in the team’s office. These students were informed that their responses would not be anonymous. The student health team had access to their answers as part of their assessment of the student’s SAP. Students in the school setting were informed that their survey responses would remain anonymous.

## Measures

### Swedish inventory of School attendance problems

The ISAP is a 48-item instrument designed to measure 13 factors related to SAPs experienced over the past 12 weeks of school. These factors include Depression, Social Anxiety, Separation Anxiety, Performance Anxiety, Agoraphobia/Panic, Somatic Complaints, School Aversion/Attractive Alternatives, Aggression, Problems with Peers, Problems with Teachers, Dislike of the Specific School, Problems within the Family, and Problems with Parents. Each item is rated in two ways: the first response relates to the symptom represented by the item (i.e., ‘How much does this apply to me?’), and the second response relates to the function of the symptom (i.e., ‘That’s why I miss school or attending school is hard for me’). Responses are given on a 4-point Likert-scale, ranging from 0 (*not true at all*) to 3 (*very much true*). Scale scores are calculated as the average of item scores.

Respondents are instructed to indicate what applies to them over the last 12 weeks of school. Factor scores are calculated by summing the item scores and dividing by the number of items in each factor, resulting in scores ranging from 0 to 3. Higher scores indicate a greater degree of the symptom (symptom scales) or the function of the symptom (function scales). Examples items include: “Before or at school/school time, I feel treated unfairly by my parents”, and “Before or at school/school time, I’m afraid of being teased or bullied by other students”. The ISAP also includes a question about the extent of school absence over the last 12 school weeks, with response options ranging from ‘not at all’ to ‘(almost) always’, categorized as: ‘not at all’, ‘sometimes’ (up to 4 school days missed), ‘often’ (5 to 12 school days missed), ‘very often’ (13 to 36 school days missed), ‘most of the time’ (37 to 48 days missed), and ‘(almost) always’ (more than 48 days missed).

To adapt the ISAP for use in Sweden (S-ISAP), the original German-language ISAP was forward translated by a teacher and back translated by a psychologist, both fluent in Swedish and German. Working collaboratively with the teacher, we made slight modifications to the translated version. The modified version was then piloted with a group of 16 Swedish-speaking youth, leading to minor wording for 19 items to enhance clarity.

## School Refusal Assessment Scale-revised

The School Refusal Assessment Scale Revised (SRAS-R) [12] measures four functions underlying youths’ school absence: (1) avoidance of objects or situations within the school setting that provoke negative affectivity (ANA); (2) escape from aversive social or evaluative situations (ESE); (3) attention-getting behavior (PA); and (4) positive tangible reinforcement (PTR). The instrument consists of 24 items rated on a 7-point Likert scale, ranging from 0 (*never*) to 6 (*always*), with 6 items related to each function. Function scale scores, which range from 0 to 6, are calculated by summing the item scores and dividing by 6 (the number of items per scale), with higher scores indicating a greater degree of the function. The SRAS-R does not specify a time period for responses. Example items include: “How often do you stay away from school because you will feel sad or depressed if you go?” and “Would you like to be home with your parents more than other kids your age would?” In the current sample, Cronbach’s alpha values for the four scales were 0.87, 0.86, 0.83, and 0.70, respectively.

## Strengths and difficulties questionnaire

The Strengths and Difficulties Questionnaire (SDQ) [20, 21] is a behavioral screening instrument that measures young people’s symptoms and positive attributes during the past six months. It consists of 25 items divided into five subscales, with five items in each: Emotional Symptoms, Conduct Problems, Hyperactivity-Inattention, Peer Problems, and Prosocial Behavior. Responses are provided on a 3-point Likert scale from 0 (*not true*) to 2 (*certainly true*), resulting in subscale scores ranging from 0 to 10, where higher scores represent a greater degree on the construct measured by the subscale. Five of the items are reversed-scored. The SDQ includes an impact supplement that measures chronicity, distress, social impairment, and burden to others. The distress and social impairment components are summed to produce an Impact rating, which ranges from 0 to 15 [22]. Example items include: “I usually do as I am told” and “I have one good friend or more”. In the present sample, Cronbach’s alpha values for the five subscales were 0.78, 0.58, 0.74, 0.58, and 0.78, respectively.

### Data analyses

Data analyses were conducted in R using the packages *lavaan* (0.6–12) [23], *psych* (version 2.2.5.) [24], and *tidyverse* (version 1.3.2) [25]. Initially, we checked for univariate and multivariate normality. In addition, respondents with 30% or more missing data were excluded, while those with less than 30% missing data were handled using the *mice* package (version 3.14.0) [26] in R.

The factor structure of the S-ISAP was tested using two separate CFAs: one for the symptom scales and another for the function scales. Estimation was based on raw data, with the Weighted Least Squares Mean and Variance adjusted (WLSMV) method used as the estimation method due to nonnormal ordinal level data [27]. Guided by the rule of thumb of having 20 cases per variable [28], we aimed for a sample size of 960.

Model fit was evaluated using five goodness-of-fit indices, as recommended by Jackson et al. [29]: the (likelihood ratio) Chi-square goodness of fit (*χ*^2^) test, the Comparative Fit Index (CFI), the Root-Mean-Square Error of Approximation (RMSEA), the Standardized Root Mean Square Residual (SRMR), and the Tucker Lewis index (TLI). A statistically non-significant Chi-square indicates an acceptable fit. SRMR should be at or below 0.08 according to Hu and Bentler [30]. RMSEA < 0.08 would indicate an acceptable fit and < 0.05 a good fit [31]. Hu and Bentler [30] suggested cut-offs for CFI and TLI at or below 0.95. Traditional, less stringent cut-off values for CFI and TLI are > 0.90 to indicate an acceptable fit [32]. Marsh and colleagues [31] suggested a norm-reference approach to choosing cut-off, where cut-offs depend on the context. Given this, we chose the cut-off of 0.90 for CFI and TLI, based on earlier CFA studies in the field of SAPs.

Internal consistency was estimated using Cronbach’s alpha [32] and McDonalds’ omega total [33], with alpha values > 0.70 considered ‘adequate’. Convergent validity was evaluated by comparing correlations between the S-ISAP subscales and subscales from other instruments measuring similar constructs. Correlation strength was categorized as small when between 0.10 and 0.29, medium when between 0.30 and 0.49, and large when above 0.50 [34]. Finally, intraclass correlation (ICC) was computed to account for the nested nature of our data, assessing potential school-level effects.

## Results

### Missing values and item level analysis of normal distribution

After removing cases with more than 30% missing data, the remaining dataset had minimal missing values, ranging from 0.0 to 0.6% per case. Specifically, 45 participants were excluded from the CFA of the symptom scales due to exceeding the 30% missing data threshold. Among the remaining participants, 341 had no missing values on the symptom scales, while 13 had between one and eight missing values across these scales. For the function scales, 71 participants were excluded from the CFA due to having than 30% missing data. Among the remaining participants, 307 had complete data for the function scales, and 21 had between one and ten missing values across the function scales.

Data were treated as ordinal. S-ISAP factors showed varying degrees of positive skewness, with most participants reporting either no or low symptoms and functions. Standardized skewness values ranged from 0.45 to 2.68 for the symptom factors, and from 1.28 to 5.31 for the function factors. Analyzing the design effect using the ICC at the item level revealed a design effect greater than 1 for 15 of the 48 symptom items and 23 out of the 48 function items (see Table 10 in the Supplementary Materials for details).

### Factor analysis

**Fig. 1:**
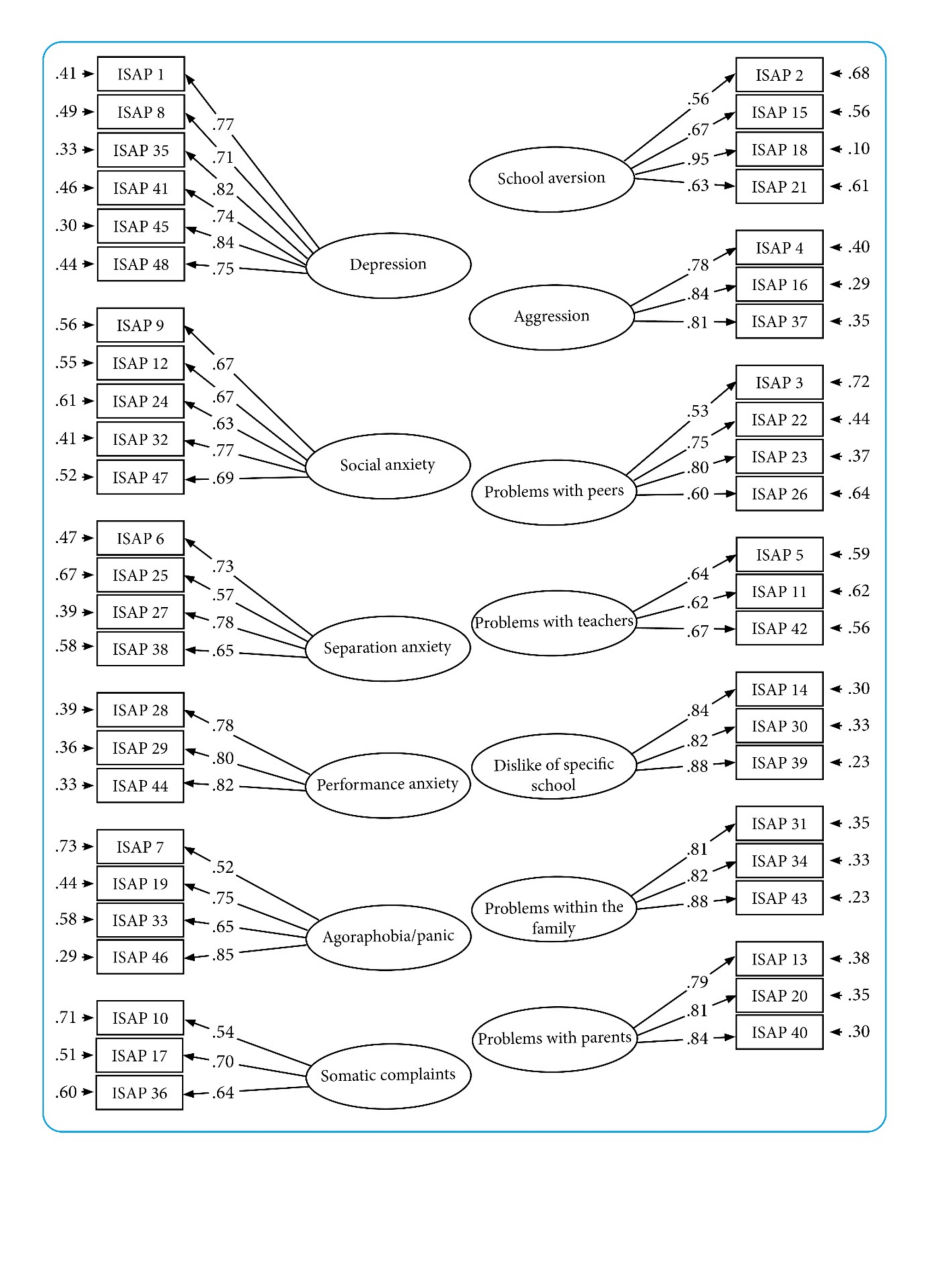
Factor loadings and error terms for the ISAP symptom scales Note: ISAP = Inventory of School Attendance Problems. Factor loadings and standardized residual variances (i.e., 1– squared factor loading). All factor loading were statistically significantly different from 0. All factors correlated (see Table 3). Correlation between factors were not included in the figure; due to the large number of factors this would result in a lack of clarity.

For the symptom scales, we observed positive correlations between items (Fig. 3 in the Supplementary Materials) and among the factors (Table 2), consistent with expectations. Items loaded onto their hypothesized factors with generally large factor loadings. All factor loadings were above 0.50, with 18 loadings falling between 0.50 and 0.70 (Fig. 1). Although the Chi-Square test (*χ*^2^ = 1381.986, *df* = 1002, *p* <.001) indicated a statistically significant misfit, the other four fit indices indicated an adequate model fit: RMSEA (0.035 [90% CI: 0.030-0.039]), SRMR (0.057), CFI (0.983), and TLI (0.981).

**Table 2:** Symptom scales. Scale intercorrelations

	(1)	(2)	(3)	(4)	(5)	(6)	(7)	(8)	(9)	(10)	(11)	(12)	(13)
(1) Depression		0.83**	0.50**	0.65**	0.68**	0.79**	0.68**	0.66**	0.70**	0.62**	0.49**	0.66**	0.56**
(2) Social anxiety			0.61**	0.61**	0.86**	0.73**	0.51**	0.49**	0.86**	0.59**	0.44**	0.58**	0.52**
(3) Separation anxiety				0.54**	0.61**	0.55**	0.40**	0.38**	0.62**	0.62**	0.42**	0.45**	0.45**
(4) Performance anxiety					0.52**	0.58**	0.48**	0.47**	0.49**	0.55**	0.32**	0.46**	0.36**
(5) Agoraphobia/panic						0.75**	0.41**	0.50**	0.79**	0.66**	0.42**	0.58**	0.59**
(6) Somatic complaints							0.47**	0.52**	0.69**	0.58**	0.46**	0.63**	0.54**
(7) School aversion								0.66**	0.45**	0.62**	0.46**	0.39**	0.38**
(8) Aggression									0.39**	0.48**	0.37**	0.50**	0.47**
(9) Problems with peers										0.74**	0.43**	0.63**	0.60**
(10) Problems with teachers											0.59**	0.57**	0.59**
(11) Dislike of specific school												0.33**	0.34**
(12) Problems within the family													0.73**
(13) Problems with parents													

*p <.05. **p <.01

**Fig. 2:**
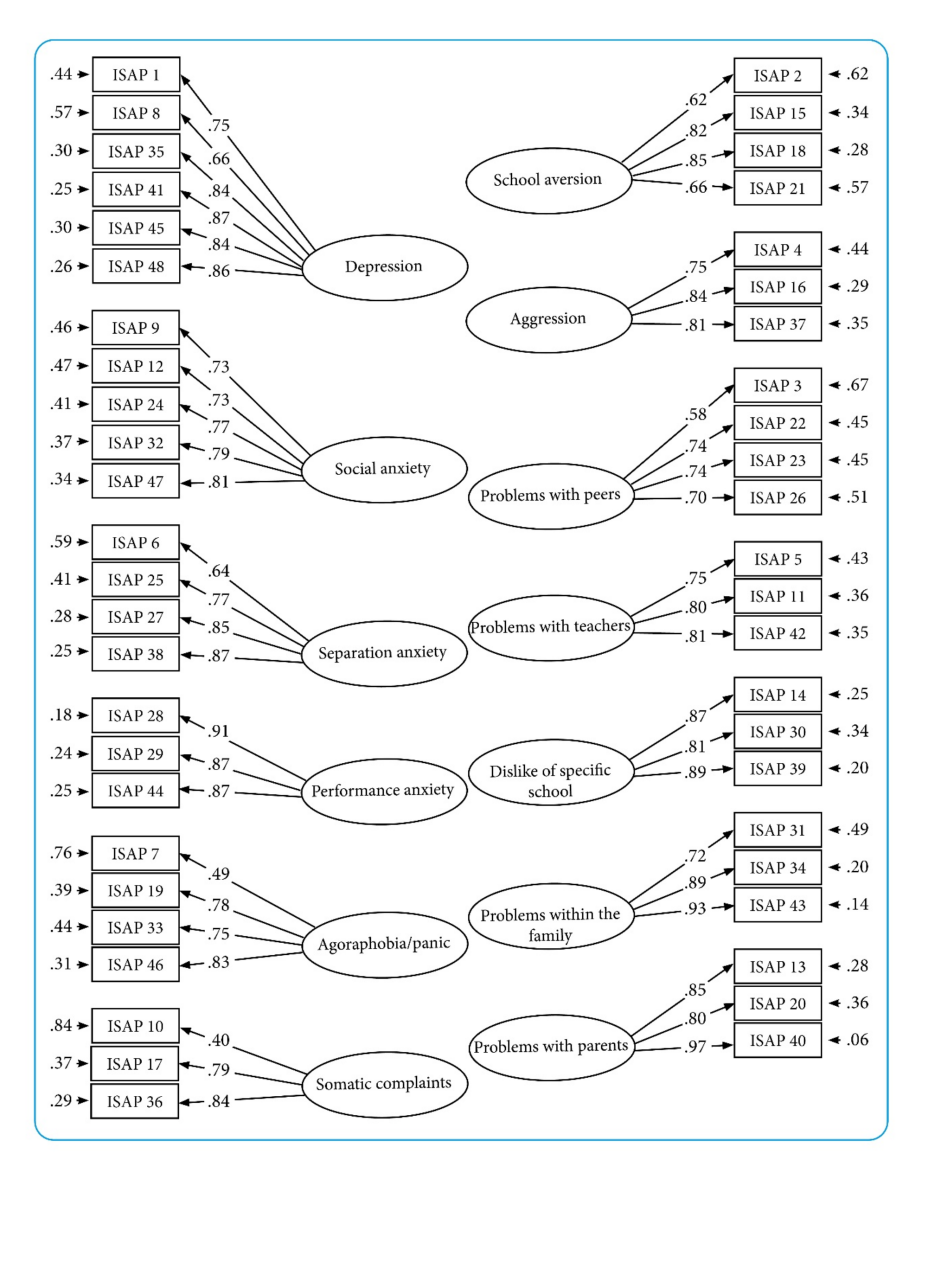
Factor loadings and error terms for the ISAP function scales Note: ISAP = Inventory of School Attendance Problems. Factor loadings and standardized residual variances (i.e., 1 – squared factor loading). All factor loading were statistically significantly different from 0. All factors correlated (see Table 4). Correlation between factors were not included in the figure; due to the large number of factors this would result in a lack of clarity.

For the function scales, items loaded onto their relevant hypothesized factors, with most factor loadings being large. Only two loadings were below 0.50, and seven were below 0.70 (Fig. 2). Positive item intercorrelations (Fig. 4 in the Supplementary Materials) and scale intercorrelations (Table 3) were observed. Although the Chi-square test (*χ*^*2*^ = 1213.742, *df* = 1002, *p* <.001) indicate a lack of adequate fit, the other fit indices supported good model fit: RMSEA = 0.023. (90% CI: 0.018–0.028), SRMR = 0.060, CFI = 0.989, and TLI = 0.988.

**Table 3:** Function scales. Scale intercorrelations

	(1)	(2)	(3)	(4)	(5)	(6)	(7)	(8)	(9)	(10)	(11)	(12)	(13)
(1) Depression		0.89**	0.62**	0.74**	0.78**	0.63**	0.85**	0.74**	0.80**	0.76**	0.70**	0.64**	0.53**
(2) Social anxiety			0.75**	0.73**	0.93**	0.60**	0.76**	0.76**	0.98**	0.78**	0.70**	0.75**	0.67**
(3) Separation anxiety				0.68**	0.72**	0.47**	0.68**	0.79**	0.79**	0.78**	0.76**	0.70**	0.80**
(4) Performance anxiety					0.70**	0.46**	0.64**	0.69**	0.73**	0.75**	0.50**	0.59**	0.57**
(5) Agoraphobia/panic						0.65**	0.62**	0.70**	0.96**	0.79**	0.67**	0.85**	0.79**
(6) Somatic complaints							0.58**	0.45**	0.65**	0.49**	0.60**	0.47**	0.41**
(7) School aversion								0.87**	0.71**	0.80**	0.68**	0.55**	0.59**
(8) Aggression									0.78**	0.83**	0.65**	0.70**	0.68**
(9) Problems with peers										0.82**	0.72**	0.89**	0.82**
(10) Problems with teachers											0.68**	0.76**	0.75**
(11) Dislike of specific school												0.58**	0.56**
(12) Problems within the family													0.83**
(13) Problems with parents													

*p <.05. **p <.01

Modification indices frequently suggested cross-loadings among items from factor 2 (Social Anxiety), factor 5 (Agoraphobia/Panic), and factor 9 (Problems with Peers). Items 6, 8, 10, 15, 17, 18, and 36 displayed cross-loadings, with three items belonging to factor 6 (Somatic Complaints) and two belonging to factor 7 (School Aversion). Covarying residuals were uncommon, except for item 17 (on the function part), which shared variance with items 10, 36, and 8. Incorporating these modifications would result in only minor changes to the model, and the Chi-square value would remain significant.

### Internal consistency

The internal consistency of the 13 factors within the symptom scales, measured by Cronbach´s alpha, ranged from 0.65 to 0.90 (Table 4), with twelve scales classified as ‘adequate’ and one (Somatic Complaints) as ‘inadequate’. When assessed using omega total, internal consistency ranged from 0.65 to 0.94. For the function scales, Cronbach´s alpha values ranged from 0.70 to 0.91 (Table 4), indicating ‘adequate’ consistency for all 13 scales. Omega total values for the function scales ranged from 0.74 to 0.95, with Somatic Complaints again showing the lowest alpha and omega values.

**Table 4:** Descriptives, internal consistency, and correlation between symptom and function scales

	Symptom							Function					
	*M*	*SD*	95% CI	Md	*α*	*ω*_*t*_	S-F ^a^	*M*	*SD*	95% CI	Md	*α*	*ω*_*t*_
(1) Depression	0.83	(0.76)	[0.75, 0.91]	0.67	0.90	0.94	0.69**	0.42	(0.68)	[0.34, 0.50]	0	0.91	0.95
(2) Social anxiety	0.58	(0.65)	[0.50, 0.66]	0.40	0.82	0.86	0.64**	0.27	(0.54)	[0.21, 0.33]	0	0.88	0.90
(3) Separation anxiety	0.50	(0.62)	[0.44, 0.56]	0.25	0.81	0.85	0.55**	0.17	(0.46)	[0.11, 0.23]	0	0.87	0.90
(4) Performance anxiety	1.17	(0.89)	[1.07, 1.27]	1.00	0.84	0.84	0.51**	0.39	(0.73)	[0.31, 0.47]	0	0.91	0.92
(5) Agoraphobia/panic	0.29	(0.55)	[0.23, 0.35]	0	0.80	0.82	0.71**	0.17	(0.44)	[0.13, 0.21]	0	0.80	0.82
(6) Somatic complaints	0.62	(0.59)	[0.56, 0.68]	0.67	0.65	0.65	0.75**	0.52	(0.65)	[0.44, 0.60]	0.33	0.70	0.74
(7) School aversion	1.13	(0.79)	[1.05, 1.21]	1.00	0.78	0.85	0.49**	0.43	(0.66)	[0.35, 0.51]	0	0.83	0.87
(8) Aggression	0.92	(0.86)	[0.82, 1.12]	0.67	0.85	0.85	0.47**	0.25	(0.57)	[0.19, 0.31]	0	0.84	0.85
(9) Problems with peers	0.38	(0.59)	[0.32, 0.44]	0	0.76	0.80	0.72**	0.21	(0.48)	[0.15, 0.27]	0	0.79	0.85
(10) Problems with teachers	0.49	(0.58)	[0.43, 0.55]	0.33	0.70	0.72	0.56**	0.21	(0.51)	[0.15, 0.27]	0	0.84	0.84
(11) Dislike of specific school	0.51	(0.77)	[0.43, 0.59]	0	0.89	0.89	0.61**	0.22	(0.57)	[0.16, 0.28]	0	0.89	0.89
(12) Problems within the family	0.40	(0.66)	[0.32, 0.48]	0	0.88	0.88	0.65**	0.19	(0.52)	[0.13, 0.25]	0	0.89	0.90
(13) Problems with parents	0.25	(0.54)	[0.19, 0.31]	0	0.84	0.85	0.54**	0.10	(0.38)	[0.06, 0.14]	0	0.91	0.91

Note. *M* = mean, *SD* = standard deviation, CI = Confidence interval, Md = Median, *α* = Cronbach’s alpha, *ω*_*t*_ = McDonalds’ Omega total

^a^ Correlation between the symptom and the function scale

*p <.05. **p <.01

### Convergent validity

The S-ISAP symptom scales Depression, Social Anxiety, Agoraphobia/Panic, and Somatic Complaints showed large positive correlations with the Emotional Symptoms scale of the SDQ (Table 5). Separation Anxiety and Performance Anxiety had medium correlations with the Emotional Symptoms scale. The S-ISAP scale Problems with Peers had a large correlation with the Peer Problems subscale of the SDQ, while Aggression had large correlations with the SDQ’s Conduct Problems and Hyperactivity subscales.

**Table 5:** Correlations of the ISAP symptoms scales with SDQ and extent of school absenteeism

	SDQ				Extent of school absenteeism ^a^
	Emotional symptoms	Conduct problems	Hyper-activity	Peer problems	
(1) Depression	0.91**	0.66**	0.51**	0.56**	0.37**
(2) Social anxiety	0.83**	0.47**	0.36**	0.67**	0.37**
(3) Separation anxiety	0.42**	0.42**	0.31**	0.44**	0.16**
(4) Performance anxiety	0.74**	0.32**	0.26**	0.29**	0.20**
(5) Agoraphobia/panic	0.71**	0.53**	0.38**	0.54**	0.23*
(6) Somatic complaints	0.87**	0.43**	0.42**	0.44**	0.43**
(7) School aversion	0.54**	0.72**	0.49**	0.37**	0.33**
(8) Aggression	0.56**	0.95**	0.65**	0.29**	0.24**
(9) Problems with peers	0.67**	0.36**	0.22**	0.74**	0.33**
(10) Problems with teachers	0.49**	0.68**	0.40**	0.46**	0.24**
(11) Dislike of specific school	0.37**	0.59**	0.40**	0.46**	0.35**
(12) Problems within the family	0.60**	0.49**	0.32**	0.33**	0.13*
(13) Problems with parents	0.45**	0.55**	0.34**	0.35**	0.13

Note. SDQ = Strengths and Difficulties Questionnaire

^a^ Last 12 weeks

*p <.05. **p <.01

For the function scales (Table 6), large correlations were observed between the S-ISAP Depression scale and the SRAS-R scales ANA, ESE, and PA. Social Anxiety on the S-ISAP also had large correlations with ANA, ESE, and PA. Performance Anxiety, Agoraphobia/Panic, and Somatic Complaints on the S-ISAP correlated with ANA, and Agoraphobia/Panic on the S-ISAP correlated with ESE. The correlation between Aggression and PTR was 0.33 and not statistically significant. Problems with Peers correlated at 0.58 and 0.79 with ANA and ESE, respectively. The largest correlations with the SDQ Impact Factor were observed for Depression, Social Anxiety, Agoraphobia/Panic, and Problems with Peers.

**Table 6:** Correlations of the ISAP function scales with SDQ impact factor, SRAS-R, and extent of school absenteeism

	SDQ	SRAS-*R*				Extent of school absenteeism ^a^
	Impact factor	ANA	ESE	PA	PTR	
(1) Depression	0.67**	0.77**	0.69**	0.55**	0.26	0.43**
(2) Social anxiety	0.57**	0.62**	0.77**	0.54**	0.26	0.33**
(3) Separation anxiety	0.32**	0.35**	0.47**	0.64**	0.33	0.13**
(4) Performance anxiety	0.47**	0.58**	0.57**	0.55**	0.33**	0.20**
(5) Agoraphobia/panic	0.54**	0.47**	0.60**	0.44**	0.22	0.15*
(6) Somatic complaints	0.46**	0.51**	0.47**	0.47**	0.22	0.42**
(7) School aversion	0.44**	0.50**	0.51**	0.48**	0.34	0.37**
(8) Aggression	0.39**	0.41**	0.51**	0.49**	0.33	0.21**
(9) Problems with peers	0.54**	0.58**	0.79**	0.52**	0.32	0.23**
(10) Problems with teachers	0.45**	0.45**	0.50**	0.51**	0.27	0.19*
(11) Dislike of specific school	0.37**	0.48**	0.58**	0.49**	0.32*	0.36**
(12) Problems within the family	0.45**	0.38**	0.51**	0.35**	0.27*	0.04
(13) Problems with parents	0.35*	0.26*	0.47*	0.36**	0.21	0.04

Note. SDQ = Strengths and Difficulties Questionnaire, SRAS-R = School Refusal Assessment Scale – Revised, ANA = avoidance of stimuli that provoke negative affectivity, ESE = escape from aversive social and/or evaluative situation, PA = pursuit of attention, PTR = pursuit of tangible reinforcement

^a^ Last 12 weeks

*p <.05. **p <.01

The S-ISAP symptom scales showing the largest correlations with the Extent of School Absenteeism were Somatic Complains, Depression, Social Anxiety, Dislike of Specific School, School Aversion, and Problems with Peers. The S-ISAP function scales that had the largest correlations with Extent of School Absenteeism were Depression, Somatic Complaints, School Aversion, Dislike of Specific School, and Social Anxiety.

## Discussion

This study addressed the psychometric properties of a Swedish version of the ISAP within an adolescent school sample. Specifically, we assessed the instrument’s factor structure, internal consistency, and convergent validity. We employed a CFA to test the hypothesized 13-factor model and evaluated internal consistency using Cronbach’s alpha and omega. Convergent validity was evaluated through correlations between constructs measured by the S-ISAP and related constructs measured by the SRAS-R and SDQ.

The findings from the factor analyses provided robust support for the hypothesized 13-factor model. First, positive correlations were observed among S-ISAP items. Second, items loaded appropriately on their theoretically relevant factors, with adequate factor loadings. Additionally, the model fit indices were satisfactory. Consistent with the original ISAP study [15], our results indicated acceptable Cronbach’s alpha and omega values. Regarding convergent validity, the S-ISAP demonstrated medium to large correlations with the SRAS-R and SDQ subscales, suggesting that the instrument effectively captured similar or related constructs. However, in some cases, the association between factors could have been underestimated due to suboptimal reliability.

Overall, the factor loadings and internal consistencies within the factors were consistently large in our analysis. Notably, none of the factor loadings fell below the threshold of 0.30, and the majority (94%) exceeded 0.60. Of the 26 scales tested, only one exhibited alpha and omega values below 0.70, indicating satisfactory internal consistency for nearly all scales. Although the Chi-square test indicated a non-adequate fit, the other four fit indices supported a good or adequate model fit for both the symptom and the function scales. The similar model fit observed between the symptom scales and function scales is likely due to the large correlations observed between the symptom and function scales (see association between symptom and function responses at the item level in Tables 8 and 9 in the Supplementary Materials).

Analysis of potential sources of misfit using modification indices revealed several cross-loadings. Specifically, the Somatic Complaints factor posed issues, with all three items showing cross-loadings, relatively low factor loadings, and lower alpha and omega total values compared to the other factors. Another potential source of misfit was the School Aversion factor, which contained three items with cross-loadings.

This study differs from the original ISAP study by Knollmann and colleagues [15] in several key ways. First, we evaluated a Swedish version of the ISAP, whereas the original version used the German version. Second, while the original research was based on a clinical sample, our study used a school-based community sample, incorporating students from regular classroom settings and those visiting the school health team in order to include students with higher levels of absence. Third, our participant age range was limited to early to middle adolescents (12–16 years old), compared to the broader age range (8–19 years old) in the original study. Fourth, we employed CFA to test the factor structure of the S-ISAP, in contrast to Knollmann and colleagues’ [15] use of PCA, which assumes uncorrelated factors. Lastly, instead of combining the symptom and function scales, we conducted separate CFAs for each.

Given these differences and the complexity of the ISAP instrument, which encompasses 13 factors, our analyses were both theoretically and empirically driven. It is important to note that this study represents a preliminary test of the instrument in a new context – the Swedish school setting with a sample of school-going adolescents. Our goal was to document the initial use and evaluation of the instrument in this context.

The findings from this initial study testing the ISAP in a Swedish non-clinical context generally support its use as a screening instrument for emerging SAPs, particularly within a sample similar to the one used in this study. Nevertheless, several limitations should be considered. First, the sample size and variability in the sampling strategy (i.e., recruiting all students in a school, students from select classes, or individuals referred to the student health team) could introduce selection biases. Although our sample was larger than that of the original ISAP study, an even larger sample would have been preferable to better detect potential model misspecifications, given the model’s complexity and the number of items per factor [35–37]. Additionally, variation the participation across different schools presents challenges to the generalizability of our findings.

Results from the ICC analysis indicated a need for a multilevel CFA to account for the nested data structure. Ignoring this nested nature can result in greater model misfit, biased parameter estimates, and less accurate standard error calculations [38]. Despite these limitations, our study provides valuable insights into the potential utility of the S-ISAP in non-clinical settings and highlights the need for further research with larger, more diverse samples to strengthen the evidence base.

Further evaluations are crucial to enhance the robustness of the ISAP and deepen our understanding of its psychometric properties. Further studies could provide stronger evidence for its factor structure, internal consistency, and convergent validity. Given the ISAP’s relative novelty, refining the instrument by clarifying construct definitions and revising items [39] could address issues identified in both our study and the original research by Knollmann and colleagues [15]. Exploring alternative, simpler models, and considering different models for the symptom and function scales, could also advance scale development.

Establishing norms and cut-off values is crucial for ensuring the ISAP’s sensitivity and specificity when using to screen for SAPs, aiding in early detection. While these enhancements are important, it is also essential to recognize that other factors such as learning difficulties may influence SAPs [40], highlighting the need for ongoing adaptation of the instrument. Nevertheless, the ISAP remains a valuable tool for practitioners due to its broad scope, separation of symptoms and functions, and design for detecting emerging SAPs. Importantly, no single questionnaire, including the ISAP, should be used as the sole means of assessment when young people display SAPs [40]. Instead, questionnaires should be integrated into a comprehensive assessment battery that includes pedagogical evaluations, interviews, and health assessments [41].

## Conclusion

This study provides preliminary evidence supporting the reliability and validity of the Swedish version of the ISAP within a community school-based sample of adolescents. The ISAP holds significant potential as a tool for exploring the diverse reasons underlying SAPs. By measuring reasons and risk factors for non-attendance with 13 symptom factors and 13 function factors, the ISAP provides a relatively comprehensive framework for researchers seeking to understand both emerging and established SAPs. Beyond research, the ISAP also offers valuable insights to inform intervention planning. Although the instrument generally performed well, some model issues were identified, and sources of misfit were explored. Further research is needed to strengthen its applicability in similar settings and evaluate its use in clinical contexts.

## Electronic supplementary material

Below is the link to the electronic supplementary material.

Supplementary Material 1

## Data Availability

The ISAP is free to download from https://www.insa.network. It is currently available in English, Finnish, German, and Swedish.

## Footnotes

1 A team comprising a school psychologist, school counsellor, school nurse, and teachers for special education needs.
