# Supplementary material for: Reliability and validity of the Swedish version of the inventory of school attendance problems (ISAP)
Source: Eur Child Adolesc Psychiatry. 2024 Nov 28;34(7):2069–82. doi: 10.1007/s00787-024-02618-0 (PMC12334511; doi:10.1007/s00787-024-02618-0)
Supplement: Supplementary file 1 — Supplementary Material 1 [file 787_2024_2618_MOESM1_ESM.docx]

**Journal name:** *European Child & Adolescent Psychiatry*

**Reliability and Validity of the Swedish Version of the Inventory of School Attendance Problems (ISAP)**

**Authors**

Johan Strömbeck, David Heyne, Laura Ferrer-Wreder, Katarina Alanko

Corresponding author

Johan Strömbeck, Åbo Akademi University, Faculty of Arts, Psychology and Theology

Fabriksgatan 2, 20500 Åbo, Finland.

E mail address: [johan.strombeck@abo.fi](about:blank)

Tel.: +46 (0)739 84 37 67

**Supplementary Materials**

**Pilot study**

It took the respondents 10-40 minutes (*M* = 18.3, *SD* = 7.7) to answer the ISAP. 10 respondents read the instructions in ISAP, and no one did not. Further, 9 of the 10 respondents answered that they understood the instructions. The items were considered understandable; only in 5 of the 48 items a small number of respondents (1-2) respondents answered that they did not understand the question. 7 items contained difficult words according to a few respondents (1-2), and 5 items contained difficult words according to several of the respondents (6-10).

After the pilot minor changes was made in 14 items to make the sentences more understandable, for instance in item 33, where 10 of the 16 participants commented on the more difficult word “*genant*” and was for that reason exchanged to “*pinsamt*”, a word that is more commonly used amongst youth today. Further, major changes were made in item 15, 18, and 43, changes that altered the meaning of the item. The first of these, item 15, was changed from “would I rather want to do something outside than to be in school” to “would I rather want to do something outside of school”, changing the meaning from wanting to be outside (as opposed to being inside), to not wanting to be in school (which could be both outside and inside, not just in school). These alterations were later accepted by MK.

The layout of ISAP was generally accepted by the participants; “easy to understand”, “clear”, but one respondent found it difficult, and did not at first notice that one should use both columns (i.e., both the left column for symptoms and the right for function).

**Table 7**

*Age distribution*

|  | *N* |
| --- | --- |
| *Age* |  |
| 12 Years Old | 5 |
| 13 Years Old | 38 |
| 14 Years Old | 123 |
| 15 Years Old | 155 |
| 16 Years Old | 78 |
| TOTAL | 399 |

*Note*. *N* = number of subjects.

On the item level, 98.6 % of those who 0 (*not true at all*) on the symptom part of an item also answered 0 (*not true at all*) on the function part of that item. Association between symptom part and function part of an item are displayed in Table 8. For half of the items, correlation between the symptom and function part of the item increased when “0” answers were removed, and for the other half correlation decreased.

**Table 8**

*Pattern of missingness. Association between symptom and function parts of the items, with and without responses “0”*

| Item no | S-F | S-F  ”0” removed |
| --- | --- | --- |
| 1 | 0,61 | 0,42 |
| 2 | 0,33 | 0,42 |
| 3 | 0,61 | 0,51 |
| 4 | 0,39 | 0,27 |
| 5 | 0,54 | 0,71 |
| 6 | 0,46 | 0,51 |
| 7 | 0,60 | 0,62 |
| 8 | 0,58 | 0,52 |
| 9 | 0,70 | 0,60 |
| 10 | 0,76 | 0,45 |
| 11 | 0,48 | 0,57 |
| 12 | 0,58 | 0,75 |
| 13 | 0,39 | 0,67 |
| 14 | 0,59 | 0,63 |
| 15 | 0,32 | 0,52 |
| 16 | 0,37 | 0,60 |
| 17 | 0,58 | 0,57 |
| 18 | 0,52 | 0,60 |
| 19 | 0,68 | 0,43 |
| 20 | 0,49 | 0,50 |
| 21 | 0,63 | 0,71 |
| 22 | 0,69 | 0,62 |
| 23 | 0,69 | 0,66 |
| 24 | 0,59 | 0,56 |
| 25 | 0,54 | 0,50 |
| 26 | 0,61 | 0,53 |
| 27 | 0,70 | 0,87 |
| 28 | 0,46 | 0,46 |
| 29 | 0,52 | 0,55 |
| 30 | 0,53 | 0,77 |
| 31 | 0,54 | 0,50 |
| 32 | 0,59 | 0,48 |
| 33 | 0,57 | 0,53 |
| 34 | 0,61 | 0,40 |
| 35 | 0,61 | 0,65 |
| 36 | 0,68 | 0,32 |
| 37 | 0,45 | 0,39 |
| 38 | 0,49 | 0,39 |
| 39 | 0,57 | 0,81 |
| 40 | 0,61 | 0,35 |
| 41 | 0,57 | 0,78 |
| 42 | 0,86 | 0,63 |
| 43 | 0,59 | 0,63 |
| 44 | 0,42 | 0,66 |
| 45 | 0,63 | 0,69 |
| 46 | 0,75 | 0,63 |
| 47 | 0,61 | 0,42 |
| 48 | 0,59 | 0,77 |

Differences between answers on the symptom and function parts of the items (Table 9). “1” means that the answer on the function part of the item were 1 point *higher* than the answer on the symptom parts. “-1” means that the answer on the function part of the item were 1 point *lower* than the answer on the symptom parts.

**Table 9**

*Difference between the symptom and function level answers*

| Item no | -3 | -2 | -1 | 0 | 1 | 2 | 3 |
| --- | --- | --- | --- | --- | --- | --- | --- |
| 1 | 8 | 20 | 91 | 211 | 13 | 2 | 2 |
| 2 | 39 | 67 | 115 | 114 | 5 | 4 | 1 |
| 3 | 1 | 8 | 51 | 270 | 10 | 2 | 0 |
| 4 | 32 | 56 | 121 | 123 | 9 | 1 | 0 |
| 5 | 5 | 9 | 57 | 265 | 5 | 0 | 1 |
| 6 | 12 | 24 | 87 | 516 | 2 | 1 | 0 |
| 7 | 3 | 5 | 18 | 308 | 5 | 0 | 0 |
| 8 | 19 | 41 | 117 | 153 | 8 | 2 | 0 |
| 9 | 1 | 9 | 41 | 278 | 6 | 2 | 1 |
| 10 | 0 | 2 | 39 | 251 | 26 | 19 | 1 |
| 11 | 7 | 32 | 111 | 178 | 5 | 1 | 0 |
| 12 | 5 | 9 | 60 | 251 | 4 | 1 | 0 |
| 13 | 7 | 9 | 43 | 265 | 4 | 2 | 1 |
| 14 | 6 | 14 | 50 | 252 | 5 | 1 | 0 |
| 15 | 35 | 51 | 107 | 130 | 5 | 3 | 0 |
| 16 | 19 | 34 | 95 | 177 | 3 | 3 | 0 |
| 17 | 7 | 16 | 89 | 199 | 14 | 4 | 3 |
| 18 | 15 | 34 | 105 | 166 | 8 | 2 | 0 |
| 19 | 3 | 8 | 34 | 277 | 6 | 1 | 0 |
| 20 | 2 | 6 | 38 | 280 | 3 | 0 | 0 |
| 21 | 7 | 15 | 60 | 237 | 12 | 0 | 0 |
| 22 | 3 | 9 | 44 | 268 | 4 | 1 | 0 |
| 23 | 1 | 8 | 37 | 276 | 5 | 1 | 0 |
| 24 | 16 | 44 | 93 | 173 | 4 | 0 | 0 |
| 25 | 6 | 15 | 91 | 212 | 3 | 0 | 0 |
| 26 | 5 | 11 | 29 | 278 | 4 | 1 | 1 |
| 27 | 0 | 3 | 24 | 301 | 2 | 0 | 0 |
| 28 | 19 | 49 | 108 | 148 | 3 | 1 | 0 |
| 29 | 20 | 35 | 121 | 145 | 5 | 1 | 0 |
| 30 | 10 | 19 | 43 | 253 | 3 | 0 | 0 |
| 31 | 1 | 15 | 65 | 238 | 4 | 1 | 1 |
| 32 | 3 | 12 | 45 | 264 | 3 | 0 | 0 |
| 33 | 1 | 5 | 35 | 283 | 2 | 0 | 1 |
| 34 | 1 | 9 | 47 | 262 | 5 | 2 | 1 |
| 35 | 7 | 21 | 72 | 224 | 4 | 0 | 1 |
| 36 | 1 | 10 | 24 | 281 | 6 | 2 | 2 |
| 37 | 9 | 27 | 74 | 212 | 2 | 2 | 1 |
| 38 | 5 | 11 | 81 | 225 | 2 | 0 | 1 |
| 39 | 9 | 12 | 52 | 250 | 3 | 0 | 1 |
| 40 | 1 | 3 | 24 | 295 | 2 | 1 | 1 |
| 41 | 4 | 18 | 74 | 228 | 3 | 0 | 1 |
| 42 | 0 | 3 | 11 | 310 | 3 | 1 | 0 |
| 43 | 1 | 16 | 50 | 253 | 6 | 0 | 0 |
| 44 | 24 | 48 | 112 | 141 | 5 | 0 | 0 |
| 45 | 5 | 24 | 93 | 202 | 3 | 1 | 0 |
| 46 | 2 | 11 | 38 | 273 | 4 | 0 | 0 |
| 47 | 6 | 17 | 69 | 229 | 6 | 0 | 0 |
| 48 | 4 | 15 | 60 | 243 | 7 | 0 | 1 |

**Table 10**

*Design effect*

| Item no | Symptom | Function |
| --- | --- | --- |
| 1 | 0 | 13,6 |
| 2 | 0 | 0,3 |
| 3 | 0,2 | 1,6 |
| 4 | 0 | 0,1 |
| 5 | 0 | 0 |
| 6 | 0 | 0 |
| 7 | 0 | 0 |
| 8 | 0,3 | 5,7 |
| 9 | 16,2 | 19,0 |
| 10 | 0 | 0 |
| 11 | 0 | 0 |
| 12 | 17,1 | 26,0 |
| 13 | 0 | 0 |
| 14 | 18,1 | 22,8 |
| 15 | 0 | 0 |
| 16 | 0 | 0 |
| 17 | 0 | 8,6 |
| 18 | 0,1 | 13,9 |
| 19 | 0 | 15,5 |
| 20 | 0 | 0 |
| 21 | 2,2 | 8,6 |
| 22 | 20,3 | 24,4 |
| 23 | 18,4 | 13,1 |
| 24 | 7,0 | 18,1 |
| 25 | 0 | 0 |
| 26 | 1,3 | 0 |
| 27 | 0 | 0 |
| 28 | 0 | 0 |
| 29 | 0,6 | 0 |
| 30 | 12,4 | 21,5 |
| 31 | 0,1 | 21,5 |
| 32 | 19,0 | 24,4 |
| 33 | 20,6 | 20,9 |
| 34 | 0,2 | 0 |
| 35 | 1,9 | 18,1 |
| 36 | 0 | 18,7 |
| 37 | 0,3 | 0,3 |
| 38 | 0 | 0,3 |
| 39 | 17,4 | 18,1 |
| 40 | 0 | 0 |
| 41 | 10,8 | 21,5 |
| 42 | 1,0 | 0,3 |
| 43 | 0 | 0 |
| 44 | 0 | 0 |
| 45 | 0 | 19,3 |
| 46 | 0 | 20,0 |
| 47 | 0 | 0 |
| 48 | 8,2 | 20,9 |

**Figure 3**

*Item intercorrelations for the ISAP symptom scales. Clustered order. Figure from corrplot version 0.92 in R*


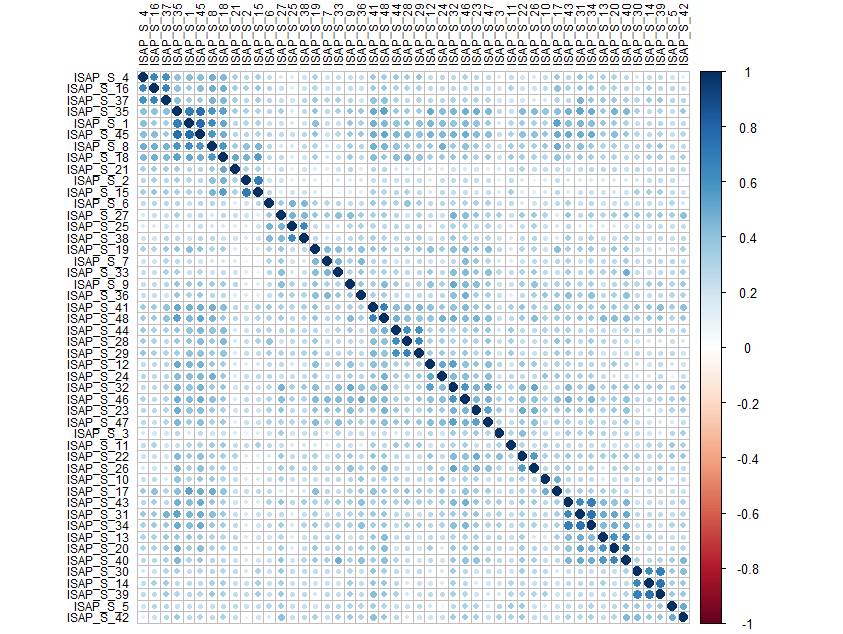


*Note*. ISAP_S = Inventory of School Attendance Problems – Symptom Scales.

**Figure 4**

*Item intercorrelations for the ISAP function scales. Clustered order. Figure from corrplot version 0.92 in R*


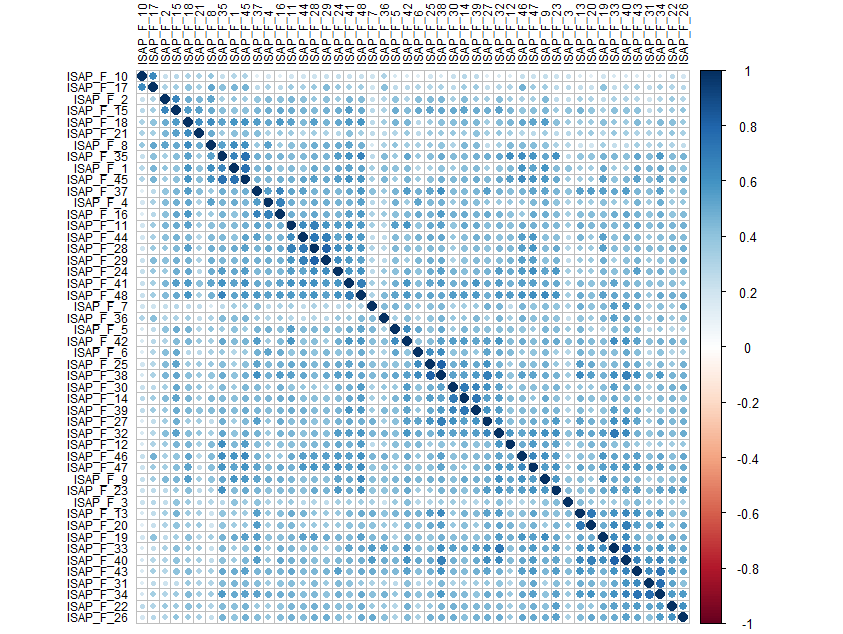


*Note*. ISAP_F = Inventory of School Attendance Problems – Function Scales.
